# Rare Contents of an Internal Hernia through a Defect of the Broad Ligament of the Uterus

**DOI:** 10.1155/2021/5535162

**Published:** 2021-05-29

**Authors:** Masafumi Takahashi, Masanori Yoshimitsu, Takuya Yano, Hitoshi Idani, Shigehiro Shiozaki, Masazumi Okajima

**Affiliations:** Department of Surgery, Hiroshima City Hiroshima Citizens Hospital, 7-33, Motomachi, Naka-ku, Hiroshima City, Hiroshima, Japan 730-8518

## Abstract

Herniation through a defect of the uterine broad ligament is a rare internal hernia that is difficult to diagnose definitively. Common hernia contents contain ileal loops. Herein, we report a rare case of internal herniation of both the ileum and fallopian tube through a defect of the broad ligament. A 52-year-old woman presented to our hospital with suprapubic pain and vomiting. She had a history of bowel obstruction following cesarean section. On abdominopelvic computed tomography, we suspected a closed-loop obstruction associated with bowel herniation in the right broad ligament. However, we could not identify an area of poor enhancement adjacent to distended small intestines. Emergency laparoscopic exploration revealed a viable ileal loop and incarcerated organ. Therefore, we switched to laparotomy that revealed the right fallopian tube as the ischemic organ. We reduced the hernia, resected necrotic right fallopian tube, and closed the defect of the broad ligament. The patient had an uneventful postoperative course. Rare hernia contents might complicate preoperative clinical diagnosis. Laparoscopy is useful for establishing a definitive diagnosis and treating broad ligament hernias.

## 1. Introduction

Internal hernias are rare causes of bowel obstruction, and internal herniation through a defect of the broad ligament of the uterus is even rarer, accounting for 4%-5% of all internal hernias [1]. Hernia contents usually include the ileum. In addition, several studies have reported constriction of organs such as the colon and fallopian tube in patients with internal hernia [2, 3]. We have recently encountered a rare case of broad ligament hernia incarcerating not only the ileum but also the fallopian tube in a middle-aged female patient. We suggest that this case might have clinical implications in the diagnosis and treatment of internal hernias.

## 2. Case Presentation

A 52-year-old woman presented to our hospital with lower abdominal pain and vomiting that started 6 hours prior to presentation. Her past medical history was significant for bowel obstruction following cesarean section. She denied having any fever or chills. There was no rebound tenderness or abdominal guarding.

**Figure 1:**
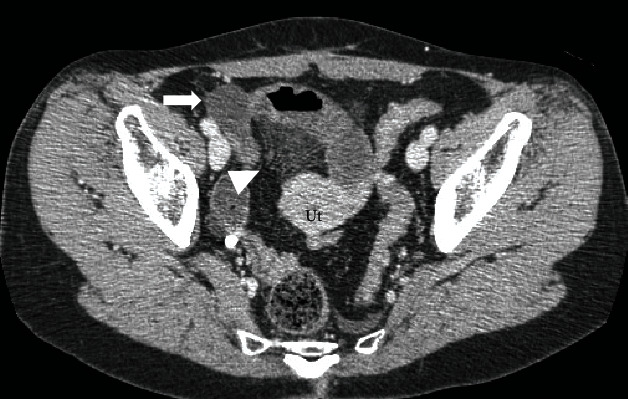
Contrast-enhanced computed tomography (CT) findings. The mesentery converges at the right side of the uterus (arrowhead). The uterus is deviated to the left. There is a spheroidal area of poor enhancement (arrow). Ut: uterus.

Laboratory test results showed an elevated white blood cell count (11,000/mm^3^). Contrast-enhanced abdominopelvic computed tomography (CT) showed a distended, fluid-filled closed loop with mesenteric fat haziness at the right side of the uterus, which was deviated to the left. The bowel wall enhancement was normal. However, there was a spheroidal area of poor enhancement adjacent to the distended small intestines, assuming it was the right ovary or ascites (Figure 1).

**Figure 2:**
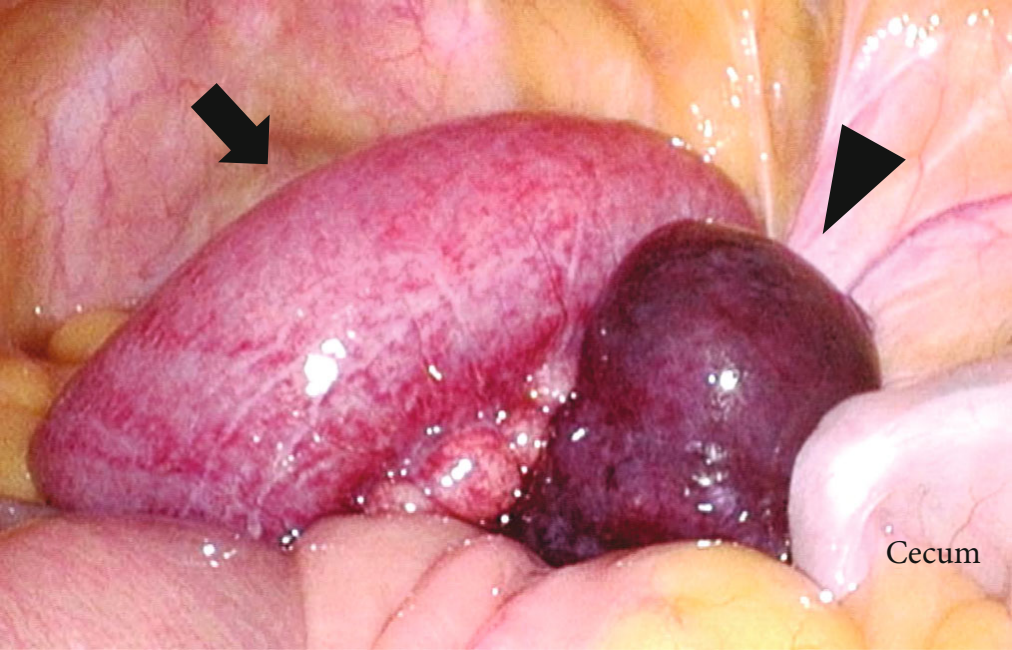
Surgical findings (laparoscopy). Within the pelvis, a viable ileal loop (arrow) and a necrotic organ are shown, suspecting it as a result of ischemia (arrowhead).

**Figure 3:**
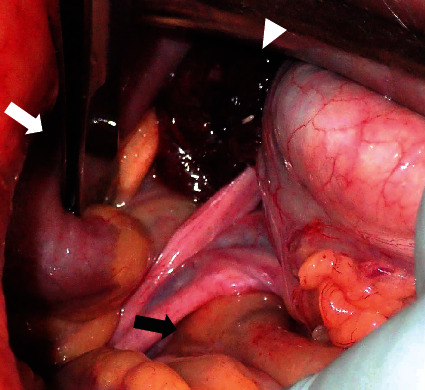
Surgical findings (laparotomy). The ileal loop (white arrow) and right fallopian tube (white arrowhead) are incarcerated, and the hernial orifice is a defect in the right broad ligament (black arrow).

Following the diagnosis of a closed-loop obstruction associated with bowel herniation in the right broad ligament of the uterus, emergency surgery was performed. Laparoscopic exploration revealed a viable ileal loop and an organ suspected to be ischemic (Figure 2). We then switched to laparotomy to enable examining this incarcerated organ. Laparotomy showed that incarceration was caused by herniation through the right broad ligament of the uterus in a posterior-to-anterior direction (Figure 3). The length of strangulated ileal loop was approximately 30 cm, which was preserved and had no ischemic changes. The organ with suspected ischemia was the enlarged ampullary portion of the right fallopian tube, which was incarcerated and gangrenous. We reduced the hernia and performed a right salpingectomy. The hernial orifice diameter was 3.0 cm, and the defect was closed with a continuous suture. The patient had a favorable postoperative course and was discharged to home on postoperative day 8.

## 3. Discussion

Internal herniation through a defect in the uterine broad ligament is extremely rare. Broad ligament defects may be congenital or acquired [4]. Congenital defects arise from spontaneous rupture of congenital cystic structures within the broad ligament. Acquired defects can be secondary to surgery, pregnancy, delivery trauma, or previous pelvic inflammatory diseases. Congenital defects are usually bilateral, while acquired defects are commonly unilateral. In recent studies, cases without a history of surgery or parturition were not rare. Therefore, evaluation of the bilateral ligaments during surgery is important.

Contents of broad ligament hernias generally include the ileum. However, previous studies have demonstrated that the colon or fallopian tubes can also be incarcerated [2, 3]. To the best of our knowledge, this is the only case report describing an incarcerated fallopian tube in a broad ligament hernia. In our case, hernia contents included the ampulla part of the right fallopian tube, which was found during surgery.

Early diagnosis of an internal herniation through a defect of the uterine broad ligament is important to enable prompt operative management before ischemic necrosis develops. However, it is generally difficult to diagnose broad ligament hernias due to nonspecific physical symptoms and laboratory tests. Recently, some reports have demonstrated that CT with consistent intravenous contrast is essential for the diagnosis of broad ligament hernias [5]. CT findings include (i) dilated small-bowel loops with air-fluid levels in the Douglas pouch; (ii) distended loops pushing against the uterus, rectum, and sigmoid colon; and (iii) congested mesentery converging at the broad ligament, which is associated with the small bowel loop. In this case, all three characteristics were found. Therefore, we suspected a broad ligament hernia. However, an incarcerated fallopian tube was not identified. On CT scanning, the spheroidal area of poor enhancement that we had assumed to be ascites was an enlarged fallopian tube. Rare herniated contents complicated the diagnosis.

Treatment of a broad ligament hernia is surgery, which should be performed immediately to decrease the duration of incarceration. Necrotic incarcerated organs must be resected. Regarding the orifice, a standard procedure is controversial. Previous studies have proposed the use of nonabsorbable sutures for the closure of a defect or a wide opening with incision of the fallopian tube and round ligament [6]. The latter is a more secure alternative than simple closure when ligaments are congenitally weak. In recent studies, simple closure has been performed more frequently.

The use of laparoscopic surgery for internal hernias, including broad ligament hernias, has increased. CT may suggest a diagnosis of a broad ligament hernia, while laparoscopy could aid in establishing a definitive diagnosis, especially in cases of rare hernial contents. Laparoscopic surgery also allows for treatment following exploration. A switch to laparotomy might be needed when laparoscopic findings suggest signs of necrotic damage to incarcerated organs or limited workspace owing to bowel distension. Establishing a definitive diagnosis with laparoscopy before a laparotomy may allow for a smaller incision. Compared with open surgery, laparoscopic surgery leads to a better postoperative course and a shorter period of hospitalization.  Table 1 presents a summary of the results of a literature review since 2018.

## 4. Conclusions

We suspected a diagnosis of internal herniation through a defect of the uterine broad ligament on preoperative CT, and laparoscopic exploration led to a definitive diagnosis. The rare herniated organs (the ileum and right fallopian tube) complicated the diagnosis.

## Figures and Tables

**Table 1:** Review of recent studies reporting broad ligament hernia.

	Authors [ref. no.]	History of surgery/parturitions	Approach	Procedure for orifice	Contents
1	Khetan et al. [7]	None	L	Closure	Small bowel
2	Takeyama et al. [8]	Two parturitions	SILS	Closure	Sigmoid colon
3	Toolabi et al. [9]	None	L	Closure	Small bowel
4	Park et al. [10]	Uterine myomectomy	L	Closure	Small bowel
5	Diémé et al. [11]	Three parturitions	L	Closure	Small bowel
6	Rajasekharan et al. [12]	Cesarean section	O	Closure	Small bowel
7	Zemour et al. [13]	Small bowel surgery	O	Closure	Small bowel
8	Fernandes et al. [14]	None	O	Closure	Small bowel
9	Fernandes et al.	None	O	Closure	Small bowel
10	Fernandes et al.	None	O	Closure	Small bowel
11	Reyes et al. [15]	Sleeve gastrectomy	O	Opening and salpingectomy	Small bowel
12	Koizumi et al. [16]	None	L	Closure	Small bowel
13	Sugishita et al. [17]	Right ovarian cyst surgery	L	Closure	Small bowel
14	Rohatgi and Joshi [3]	10^th^-day postcesarean section	L	Opening and salpingectomy	Small bowel and gangrenous fallopian tube (spontaneously reduced)
15	Hashimoto et al. [6]	Broad ligament hernia	O	Closure	Small bowel
16	Harvitkar et al. [18]	Cesarean section	L	Closure	Small bowel (spontaneously reduced)
17	Kaur et al. [19]	Cesarean section	O	Closure	Small bowel
18	Tirado-Peraza et al. [20]	None	O	Closure	Small bowel

Abbreviations: O: open; L: laparoscopic surgery; SILS: single-incision laparoscopic surgery.

## Data Availability

The datasets supporting the conclusions are included within this paper.
